# Supplementary material for: The characteristics and evolution of pulmonary fibrosis in COVID-19 patients as assessed by AI-assisted chest HRCT
Source: PLoS One. 2021 Mar 23;16(3):e0248957. doi: 10.1371/journal.pone.0248957 (PMC7987145; doi:10.1371/journal.pone.0248957)
Supplement: Attachment — Submitted filename: Response to Reviewers OF PONE-D-20-31950.docx [file pone.0248957.s005.docx]

Dec 22, 2020

Dear Editors and Reviewers:

Thank you for your letter and for the reviewers’ comments concerning our manuscript entitled “The characteristics and evolution of pulmonary fibrosis in COVID-19 patients as assessed by AI-assisted chest HRCT”. (ID: PONE-D-20-31950).

These comments are very constructive and helpful for revising our paper. We have discussed the comments carefully and have made revisions which we hope will be met with approval. They are all marked in blue in the revised version. The main answers to the reviewer’s comments are as follow:

1. Please ensure that your manuscript meets PLOS ONE's style requirements, including those for file naming.

**Answer:** Thank you for this comment. We have revised the article format according to PLOS ONE's style requirements.

2. In the ethics statement in the manuscript and in the online submission form, please provide additional information about the patient records/samples used in your retrospective study, including: a) whether all data were fully anonymized before you accessed them; b) the date range (month and year) during which patients' medical records/samples were accessed.

**Answer:** Thank you for this comment. We make sure that all data were anonymous. The patients' medical records were accessed from February 1 to June 8, 2020. We have added these to the revised version and marked in blue in page 5, line 77-78.

3. Thank you for stating the following in the Funding Section of your manuscript:

"This study was supported by grant 81770981 and 82002863 from the
National Natural Science Foundation of China and grant [2018]116 from Wuhan
Municipality Young and Middle-aged Medical Talent Cultivation Program."

We note that you have provided funding information that is not currently declared in your Funding Statement. However, funding information should not appear in the Acknowledgments section or other areas of your manuscript. We will only publish funding information present in the Funding Statement section of the online submission form.

Please remove any funding-related text from the manuscript and let us know how you would like to update your Funding Statement. Currently, your Funding Statement reads as follows:

"Unfunded studies"

Please include your amended statements within your cover letter; we will change the online submission form on your behalf.

**Answer:** Thank you for this comment. We have removed the fund information in the manuscript and added it in the cover letter. However, we can not added it in the online submission form. We would be very grateful if you could change the online submission form on our behalf.

4. PLOS requires an ORCID iD for the corresponding author in Editorial Manager on papers submitted after December 6th, 2016. Please ensure that you have an ORCID iD and that it is validated in Editorial Manager. To do this, go to ‘Update my Information’ (in the upper left-hand corner of the main menu), and click on the Fetch/Validate link next to the ORCID field. This will take you to the ORCID site and allow you to create a new iD or authenticate a pre-existing iD in Editorial Manager. Please see the following video for instructions on linking an ORCID iD to your Editorial Manager account: <https://www.youtube.com/watch?v=_xcclfuvtxQ>

**Answer:** Thank you for this comment. We have already applied ORCID iD ([0000-0002-1216-004X](javascript:popup_orcidDetail('https://orcid.org','0000-0002-1216-004X');)).

5. Your ethics statement should only appear in the Methods section of your manuscript. If your ethics statement is written in any section besides the Methods, please delete it from any other section.

**Answer:** Thank you for this comment. We've removed ethics statement in any other section.

**Reviewer 1:**

Specific comments:

**1.** In the abstract, you repeated the phrase “IL-6 and albumin are independent risk factors for pulmonary fibrosis” twice.
**Answer:** Thank you for this comment. We have revised it and marked in blue in page 2, line 12.

**2.** there is a typo, for it is written “SARA-CoA-2” instead of “SARS-CoV-2”.

**Answer:** Thank you for this comment. We have revised it and marked in blue in page 5, line 69.

**3.** milliampere-second is written in one word.

**Answer:** Thank you for this comment. We have revised it and marked in blue in page 6, line 89-90.

4. “anterior endobasal segment” is not a term commonly used in the anatomy of lung segments. Did you mean medial segment of the lower lobe? Please, correct.

**Answer:** Thank you for this comment. “anterior endobasal segment” mean“anterior and basal segmental”. We have revised it and marked in blue in page 6, line 97-99.

5. you say that 248 patients were enrolled, clearly a refuse.

**Answer:** Thank you for this comment. We have revised it and marked in blue in page 9, line 157.

**6.** there is an imprecision that I would suggest you to correct. In fact, you correctly reported that SARS-CoV-2 is similar for the 85% of genome to a bat coronavirus, but after that you included on parenthesis SARS-CoV: SARS-CoV-2, instead, share 85% of its genome with bat coronavirus bat-SL-CoVZC45 (Xu J, Zhao S, Teng T, Abdalla AE, Zhu W, Xie L, Wang Y, Guo X. Systematic Comparison of Two Animal-to-Human Transmitted Human Coronaviruses: SARS-CoV-2 and SARS-CoV. Viruses. 2020 Feb 22;12(2):244. doi: 10.3390/v12020244. PMID: 32098422; PMCID: PMC7077191).

**Answer:** Thank you for this comment. We have revised SARS-CoV-2 share 85% of its genome with bat coronavirus bat-SL-CoVZC45 in the revised version and marked in blue in page 3, line 34-35.

7. you used the term “shadow” to describe many CT findings associated with COVID-19 pneumonia. This term it is not used by other radiologists in an international setting: I suggest you to change it with the common used “linear opacities” and honeycombing. I did not understand the term “mesh shadows”: could you explain it better?
**Answer:** Thank you for this comment. “shadow”should be “linear opacities”. “mesh shadows”should be “reticulation”. We have revised it and marked in blue in the Manuscript and Revised Manuscript with Track Changes.

8. you cited a score for lung fibrosis proposed by Camiciottoli, followed by an apex “1”. You didn’t cite the relative paper.
**Answer:** Thank you for this comment. We have added references in the Manuscript and Revised Manuscript with Track Changes. (Camiciottoli G, Orlandi I, Bartolucci M, Meoni E, Nacci F, Diciotti S, et al Lung CT densitometry in systemic sclerosis: correlation with lung function, exercise testing, and quality of life. Chest. 2007;131(3):672-681. doi.10.1378/chest.06-1401. )

**9**. you say that patients with pulmonary fibrosis were 239, while in the rest of the text you say 237. I suggest you to correct the refuse.
**Answer:** Thank you for this comment. The patients with pulmonary fibrosis were 239. We have corrected it in the rest of the text and marked in blue in page 9, line 174.

**10.** paragraph “The relationship between the degree of pulmonary fibrosis and the clinical classification”: I suggest you to write again the initial part of the paragraph where you enlist the amount of patients with fibrosis for each group, because as it is written by now it is confusing and it is necessary to look at table 1 to understand what you meant.

**Answer:** Thank you for this comment. We have write again the paragraph and marked in blue in page 9-10, line 174-183.

**11.**In the study, you found a statistical association with the elevation of AST and severity of fibrosis. A recently published study showed a peculiar association between AST/ALT ratio, known also as de Ritis ratio, and hospital mortality in COVID-19 patients, tied also to a larger pneumonia (Zinellu A, Arru F, De Vito A, Sassu A, Valdes G, Scano V, Zinellu E, Perra R, Madeddu G, Carru C, Pirina P, Mangoni AA, Babudieri S, Fois AG. The De Ritis ratio as prognostic biomarker of in-hospital mortality in COVID-19 patients. Eur J Clin Invest. 2020 Oct 11:e13427. doi: 10.1111/eci.13427. Epub ahead of print. PMID: 33043447; PMCID: PMC7646002). I suggest you to read this work and evaluate if de Ritis ratio was also associated with more severe pulmonary fibrosis. Moreover, another study found an association with others laboratory findings and combined indexes, and I suggest you to have a look for a comparison: Paliogiannis P, Zinellu A, Scano V, Mulas G, De Riu G, Pascale RM, Arru LB, Carru C, Pirina P, Mangoni AA, Fois AG. Laboratory test alterations in patients with COVID-19 and non COVID-19 interstitial pneumonia: a preliminary report. J Infect Dev Ctries. 2020 Jul 31;14(7):685-690. doi: 10.3855/jidc.12879. PMID: 32794454.

**Answer:** Thank you for this comment. It is a very good suggestion.We have found that CRP/albumin ratio, platelet/ lymphocyte ratio was associated with pulmonary fibrosis. We have included these results in the revised version and marked in blue in page 13.

12. It is a great pity that after all the good analysis you conducted you didn’t specify the category of patients who underwent CT at 30, 60 and 90 days from discharge: it would be very interesting to know if they were all severe cases or belonged to different groups. I suggest you to specify it. I would also like to know why only few patients had a new CT scan: were they symptomatic? Did symptoms mismatch CT findings? Why you suggest the use pf antibiotics in patients showing residual interstitial abnormalities after 90 days?
    The abstract is concise and it is representative of the full article, and discussion and conclusion are coherent with the study and data shown. The paper is generally well-written, but there are many phrase constructions atypical for English grammar which probably reflects a literal translation from your native language. I suggest an overall revision of the language, preferably from a mother tongue one.
    **Answer:** Thank you for these comment.

They belonged to different groups.We have revised it and marked in blue in page 14 line 241-243

All of the patients are required to undergo a chest CT scan after discharge in accordance with the guidelines, but many of them worry about radiation in chest CT. So only few patients had a new CT scan. And their symptoms were matched with CT findings.

As for the usage of antibiotics, we have made a mistake. It should be anti-fibrosis treatment. We have revised it and marked in blue in page 17, line 299.

As for the English grammar, we also know this is a very important issue. The revised manuscript has been proofread by an English-speaking professional with science background at Springer Nature Author Services. The certificate is uploaded as an attachment.

**Reviewer 2:**

1. “Pulmonary fibrosis is a serious complication of viral pneumonia, which often leads to dyspnea and impaired lung function”: I suggest “pulmonary fibrosis can occur as a serious …”

**Answer:** Thank you for this comment.We have revised it and marked in blue in page 3, line 24 .

**2.** I suggest: “Chest x-rays and high-resolution-CT (citing Severity of lung involvement on chest X-rays in SARS-coronavirus-2 infected patients as a possible tool to predict clinical progression: an observational retrospective analysis of the relationship between radiological, clinical, and laboratory data. Baratella E, Crivelli P, Marrocchio C, Bozzato AM, Vito A, Madeddu G, Saderi L, Confalonieri M, Tenaglia L, Cova MA. J Bras Pneumol. 2020 Sep 21;46(5):e20200226. doi: 10.36416/1806-3756/e20200226. eCollection 2020).

**Answer:** Thank you for this comment. We have revised it and marked in blue in page 3, line 44 .

**3.**Chest CT protocol: Authors could better explain the mean radiation dose exposure during CT.

**Answer:** Thank you for this comment. The mean radiation dose during CT is 7 mGy. We have revised it and marked in blue in page 6, line 89-90.

4. Chest CT image analysis: Authors could better clarify the years of experience on chest CT images.

**Answer:** Thank you for this comment. The years of experience on chest CT images analysis included the distribution of the lesion, the location of the lesion, the number of lobes involved, the characteristics of the lesion and external involvement. We have revised it and marked in blue in page 6, line 97-99 .

**5.** “… striated shadow or mesh shadow…” Authors could explain the mean of these words.
**Answer:** Thank you for this comment. “striated shadow” should be “linear opacities”.“mesh shadow” should be” reticulation”. We have revised it and marked in blue in the Manuscript and Revised Manuscript with Track Changes.

All of the revisions are shown here. We would like to express our great appreciation to you and reviewers for comments on our paper.

Looking forward to hearing from you.

Best regards,

Shi-Ming Chen
