# Supplementary material for: The characteristics and evolution of pulmonary fibrosis in COVID-19 patients as assessed by AI-assisted chest HRCT
Source: PLoS One. 2021 Mar 23;16(3):e0248957. doi: 10.1371/journal.pone.0248957 (PMC7987145; doi:10.1371/journal.pone.0248957)
Supplement: Attachment — Submitted filename: Response to Reviewers OF PONE-D-20-31950 .docx [file pone.0248957.s006.docx]

Feb 16, 2021

Dear Editors and Reviewers:

Thank you for your letter and for the reviewers’ comments concerning our manuscript entitled “The characteristics and evolution of pulmonary fibrosis in COVID-19 patients as assessed by AI-assisted chest HRCT”. (ID: PONE-D-20-31950).

These comments are very constructive and helpful for revising our paper. We have discussed the comments carefully and have made revisions which we hope will be met with approval. They are all marked in blue in the revised version. The main answers to the reviewer’s comments are as follow:

1. The paper was revised in the correct way, although no article is mentioned in support of important laboratory prognostic indices in particular on liver function (https://doi.org/10.1111/eci.13427)

**Answer:** Thank you for this comment. This is a good suggestion.

It was reported that the AST/ALT ratio on admission was significantly associated with in-hospital mortality in COVID-19 patients[https://doi.org/10.1111/eci.13427]. In this work, we found that there were significant relationships between the levels of AST and pulmonary fibrosis. AST displays the highest activity in the liver and skeletal muscle but also occurs in several tissues, including lungs, heart muscle, kidneys, pancreas, brain, leucocyte and erythrocytes. AST is less specific for liver damage compared to ALT[https://doi.org/10.1111/eci.13427]. Therefore, COVID-19 patients with a significant increase in AST may also have large damage in other tissues, including the lungs, which may affect pulmonary fibrosis at discharge. Larger studies are required to confirm the capacity of this parameter to independently predict pulmonary damage and fibrosis in this group. We have revised it and added the article as a reference in the revised version.

2. There are some spelling errors in page 2, line 11-13. It should be as follow: The IL-6 level in the acute stage and albumin level were independent risk factors for pulmonary fibrosis. We have revised it and marked in blue in the revised version.

3. There are some spelling errors in page 12, line 214-218. It should be as follow: Patients with or without pulmonary fibrosis had statistically significant differences in age, IL-6 levels, lymphocyte %, aspartate transaminase (AST), albumin, CRP/albumin ratio, platelet/lymphocyte ratio and some other indexes (Table 3), suggesting that these abnormal clinical indicators may be related to the pulmonary fibrosis. We have revised it and marked in blue in the revised version.
4. In addition, there are some errors in table 3 and table 4 because of the translation. We have revised it and marked in blue in the revised version.

All of the revisions are shown here. We would like to express our great appreciation to you and reviewers for comments on our paper.

Looking forward to hearing from you.

Best regards,

Shi-Ming Chen
